# Bioactive properties and clinical safety of a novel milk protein peptide

**DOI:** 10.1186/1475-2891-10-99

**Published:** 2011-09-26

**Authors:** Richard B Kreider, Mike Iosia, Matt Cooke, Geoffrey Hudson, Chris Rasmussen, Helen Chen, Olof Mollstedt, Men-Hwei Tsai

**Affiliations:** 1 Exercise and Sport Nutrition Laboratory, Department of Health & Kinesiology, Texas A&M University College Station, TX 77843-4243, USA; 2 Department of Health, Exercise Science, and Secondary Education, Lee University, Cleveland, TN 37320, USA; 3 Schools of Biomedical & Health Sciences, Victoria University, Melbourne Victoria 8001, Australia; 4 School of Human Performance and Recreation, University of Southern Mississippi, Hattiesburg, MS 39406, USA; 5 Ambryx Biotechnology, Inc., Riverside, CA, 92507-2155, USA

**Keywords:** Health, Cancer, Neutrophil Lymphocyte Ratio, Insulin Sensitivity, Quality of Life

## Abstract

**Background:**

Milk protein fractions and peptides have been shown to have bioactive properties. This preliminary study examined the potential mechanisms of action and clinical safety of novel milk protein peptide (MP).

**Findings:**

A novel MP mixture inhibits the tyrosine kinase activity of epidermal growth factor receptor (EGFR), vascular endothelial growth factor receptor 2 (VEGFR2), and insulin receptor (IR) with IC_50 _of 9.85 μM, 7.7 μM, and 6.18 μM respectively. *In vitro*, this multi-kinase inhibitor causes apoptosis in HT-29 colon cancer cells, and in a *C. elegans *worm study, showed a weak but significant increase in lifespan. A six week double-blind, placebo-controlled study involving 73 healthy volunteers demonstrated that the MP mixture is safe to consume orally. All clinical blood markers remained within normal levels and no clinically significant side effects were reported. There was some evidence of improved insulin sensitivity, neutrophil-to-lymphocyte ratio (NLR), and quality of life assessment of role of physical function.

**Conclusions:**

These data in combination with the observed *in vitro *anti-cancer properties warrant further clinical studies to investigate this MP mixture as a potential clinical nutrition intervention for improving the quality of life and clinical outcomes in cancer patients.

**Trial Registration:**

NCT01412658

## Findings

Clinical nutrition products are specifically formulated nutrients to help people manage various health conditions. With recent advances in biotechnology, new techniques and tools are now available to isolate components from food that have additional health enhancing properties. Peptides are good candidates for advanced clinical nutrition and supplements since they are easily absorbed, and unlike many plant based compounds, peptides are amino acids that can be eliminated naturally by the body with less potential toxicity [1]. We have isolated a peptide mixture from regular cow's milk that preliminary basic and clinical research indicates may have some beneficial bioactive properties. The purpose of this study was to: (1) characterize the molecular mechanism of action; (2) assess the mixture's safety in healthy human subjects; and, (3) look for trends in potential improvements that can be further explored in future clinical trials in cancer patients.

## Methods

### Milk peptides

A milk peptide product AminoAct^® ^(MP) was prepared from normal cow's milk and supplied by OncoNutrition (*Riverside, CA*). A whey based peptide fraction was isolated from pasteurized skim milk using proprietary enzyme digestion and buffers. The final product was supplied in an aqueous solution. Table 1 presents the nutrient panel of the MP product examined in this report.

**Table 1:** Nutrient content of milk peptide product (per 100 ml).

Ingredient	Amount
Calories (kcal)	12.1
Fat (kcal)	3.8
Fat (g)	0.4
Saturated Fat (g)	0.16
Sodium (mg)	476
Protein (g)	2
Calcium (mg)	112
Trans Fat (g)	0
Cholesterol (mg)	0
Sugars (g)	0
Carbohydrate (g)	0
Dietary Fiber (g)	0
Iron (mg)	0
Vitamins	0

### Basic Research Trials

#### Kinase IC_50 _Profiling

The inhibitory effects of the milk peptide mixture on epidermal growth factor receptor (EGFR), vascular endothelial growth factor receptor 2 (VEGFR2), and insulin receptor (IR) kinases were performed at Reaction Biology Corporation (*Malvern, PA*). Briefly, specific kinase/substrate pairs along with required cofactors were prepared in reaction buffer; 20 mM Hepes pH 7.5, 10 mM MgCl_2_, 1 mM EGTA (ethylene glycol tetraacetic acid), 0.02% Brij35, 0.02 mg/ml BSA (bovine serum albumin), 0.1 mM Na_3_VO_4_, 2 mM DTT (dithiothreitol), 1% DMSO (dimethyl sulfoxide). Compounds were delivered into the reaction, followed 20 minutes later by addition of a mixture of ATP (*Sigma, St. Louis MO*) and ^33^P ATP (*Perkin Elmer, Waltham MA*) to a final concentration of 10 μM. Reactions were carried out at room temperature for 120 min, followed by spotting of the reactions onto P81 ion exchange filter paper (*Whatman Inc., Piscataway, NJ*). Unbound phosphate was removed by extensive washing of filters in 0.75% Phosphoric acid. After subtraction of background derived from control reactions containing inactive enzyme, kinase activity data was expressed as the percent of remaining kinase activity in test samples compared to vehicle (dimethyl sulfoxide) reactions. Ten concentrations of milk peptides, with highest at 500 μM concentration, were tested. IC_50 _values and curve fits were obtained using Prism (GraphPad Software, La Jolla, California).

#### HT-29 Colon Cancer Cell Apoptosis Assay

HT-29 human colon cancer cells were grown in a 72-well Terasaki plate until 80% confluent in McCoy's 5A medium, 10% FBS [2]. Vehicle, commercial hydrolyzed whey protein (*Thorne Research, Sandpoint*, *ID*), and MP mixture were added separately to each well in triplicates for a final concentration of 0.68 μg/μl media. Pictures were taken after 20 hr incubation.

#### *C.elegans *Lifespan Assay

The effects of the MP mixture on the lifespan of *C. elegans *were performed at the University of Utah *C. elegans *Research Core Facility (*Salt Lake City, Utah*) using previously published methods [3]. Nematode Growth Media (NGM) agar plates were prepared containing either no peptides, or 20 μg/ml, 6.66 μg/ml, 0.66 μg/ml concentrations of the MP mixture. All plates were seeded with *E. Coli *strain OP50. Experimental plates were prepared in triplicate. Approximately 10 N2 (wild type strain) worms were cultured on four standard agar plates or plates containing different dosages of MP. In addition to using N2 worms on standard plates as controls, other control plates included 40L4 mutant daf-2 (el370) and 40 L4 daf-16(m26) larvae. The mean number of surviving worms for each day was recorded, and the number of animals died on each day was calculated and the values were used in log rank tests to examine statistically significant differences. The results were simultaneously evaluated using the χ^2 ^test and values above 1.0 at a p-value of > 0.01 are considered statistically different when comparing longevity of N2 worms on standard plates versus the test sample.

### Clinical Safety Study

#### Subjects

The clinical trial was conducted as a placebo-controlled, double-blind, parallel arm repeated measures study conducted in a university setting. The protocol was reviewed and approved by a human participants internal review board (IRB) and written informed consent was obtained from all patients prior to the initiation of study. Subjects who were apparently healthy between the ages of 35 - 60 years who had body mass index (BMI) ranging between 27 and 40 with no known metabolic disorders, history of milk allergies, and/or medical conditions were eligible to participate in the study. Subjects were matched according to gender, age, and body mass and randomized to consume a placebo or the MP mixture in a free living condition. Table 2 presents participant demographics based on gender and group randomization. Eighty-one subjects volunteered to participate in this study with 73 completing the study. Two males and six females did not complete the study. Three cited time constraints with their schedules, two missed multiple testing sessions, 2 did not give a specific explanation and one participant who was in the supplement group withdrew due to gastrointestinal distress which could have been instigated by ingestion of the milk or supplement.

**Table 2:** Participant descriptive data.

Variable	Group	Male	Female	Group × Gender p-level
Age (yr)	MP	47.0 ± 8	45.8 ± 8	0.11
	P	45.2 ± 10	50.6 ± 8	
Height (cm)	MP	177 ± 11	163 ± 7	0.86
	P	176 ± 6	163 ± 8	
Weight (kg)	MP	99.1 ± 13	82.5 ± 10	0.82
	P	98.9 ± 13	83.8 ± 14	
BMI (kg/m^2^)	MP	31.7 ± 4	30.8 ± 3	0.75
	P	31.8 ± 4	31.5 ± 4	
Body Fat (%)	MP	31.1 ± 7	40.0 ± 5	0.12
	P	30.8 ± 4	43.6 ± 4	

Data are means and ± standard deviations. MP = Milk Protein. P = Placebo.

#### Supplementation protocol

The MP mixture or a flavored water and glycerol placebo were administered in three escalating dosages based on body weight over a six week period according to the supplementation schedule described in Table 3. Subjects took 1/2 dosage, mixed with 1/2 cup milk immediately after breakfast and consumed the remaining 1/2 dosage with 1/2 cup milk immediately after dinner. Supplementation compliance was monitored by having participants return empty bottles of the supplements as well as submitting a supplement log documenting when supplements were ingested.

**Table 3:** Supplementation dosing schedule (ml/d).

Weight Range	Dosage for Week 1 & 2	Dosage for Week 3 & 4	Dosage for Week 5 & 6
< 100 kg	6	12	15
101 - 109 kg	9	15	18
> 110 kg	12	18	21

#### Clinical assessments

Subjects were evaluated at baseline, 3 weeks, and 6 weeks for general health markers. The independent variable was MP supplementation. Dependent variables included energy intake; body composition; resting heart rate and blood pressure; a complete whole blood and serum clinical chemistry panel; serum insulin and leptin; measures of quality of life; and, symptoms and side effects surveys.

#### Procedures

Participants recorded all food and fluid intake for four days prior to each testing session which included three weekdays and one weekend day. Dietary inventories were reviewed by a registered dietitian and analyzed using the ESHA Food Processor (*Version 8.6*) Nutritional Analysis software (*ESHA Research Inc*., *Salem, OR*). Height and body mass were determined using a calibrated electronic scale (*Cardinal Detecto Scale Model 8430, Webb City, Missouri*). Body composition (excluding cranium) was assessed using a Hologic Discovery W (*Hologic Inc., Waltham, MA*) dual energy x-ray absorptiometer (DXA) equipped with APEX Software (*APEX Corporation Software, Pittsburg, PA*). Resting heart rate was determined by palpitation of the radial artery and blood pressure was assessed by auscultation of the brachial artery using a mercurial sphygmomanometer using standard clinical procedures [4].

Whole blood samples were analyzed for complete blood counts with platelet differentials using an Abbott Cell Dyn 3500 (*Abbott Laboratories, Abbott Park, IL*) automated hematology analyzer. Serum samples were analyzed for a complete metabolic panel using a Dade Behring Dimension RXL (*Siemans AG, Munich, Germany*) automated clinical chemistry analyzer. Coefficient of variation (range: 1.0 to 9.6%) for tests performed was similar to previously published data for these tests [5]. Serum insulin and leptin were determined using commercially available immuno-absorbent assay (ELISA) kits (*Diagnostic Systems Laboratories, Webster, TX*) using a Wallac Victor-1420 microplate reader (*Perkin-Elmer Life Sciences, Boston, MA*) according to kit specifications. Intra-assay and inter-assay coefficient of variation were 4%-7% for insulin and 2% - 8% for leptin. The homeostasis model assessment for estimating insulin resistance (HOMA_IR_) was calculated as the product of fasting glucose times fasting insulin expressed in conventional units divided by 405 [6]. Participants also completed the SF-36 Quality of Life (QOL) inventory which has been used to assess physical and mental components related to QOL [7] as well as a symptom and side effect questionnaire.

### Statistical analysis

Related variables were grouped together and analyzed by multivariate analysis of variance (MANOVA) with repeated measures using PASW Statistics 18.0.2 software (*SPSS, Chicago, IL*). Non-correlated variables were analyzed by univariate repeated measures analysis of variance (ANOVA). Delta values were calculated and analyzed on select variables by MANOVA for repeated measures to assess changes from baseline values. Data were considered statistically significant when the probability of type I error was 0.05 or less. If a significant group, treatment and/or interaction alpha was observed, Tukey's honestly significant differences post-hoc analyses were performed.

## Results

### Basic Research Findings

**Figure 1:**
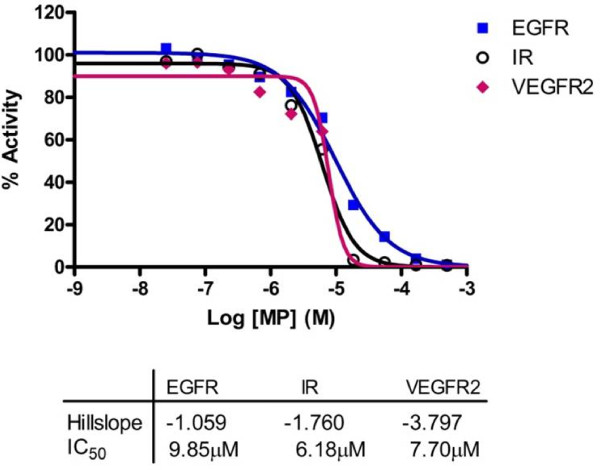
**Determination of tyrosine kinase inhibitory properties of novel milk peptide mixture MP**. The novel milk peptide mixture was screened against a panel of kinases. Specifically, the IC_50 _for inhibition of epidermal growth factor receptor (EGFR), insulin receptor (IR), and vascular epidermal growth factor receptor-2 (VEGFR2) were determined using a 10-point dose response radiolabeled assay.

**Figure 2:**
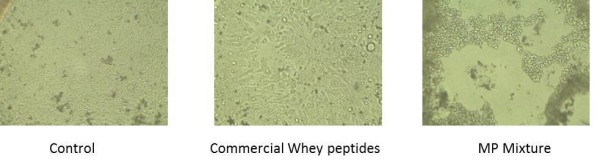
**Comparison of HT-29 human colon cancer cell death between commercially available whey peptides and the MP mixture**. HT-29 colon cancer cells were incubated with 0.68 μg/μl concentration of commercial hydrolyzed whey protein, MP peptides, and vehicle only (control). Pictures were taken after 20 hours of incubation.

**Figure 3:**
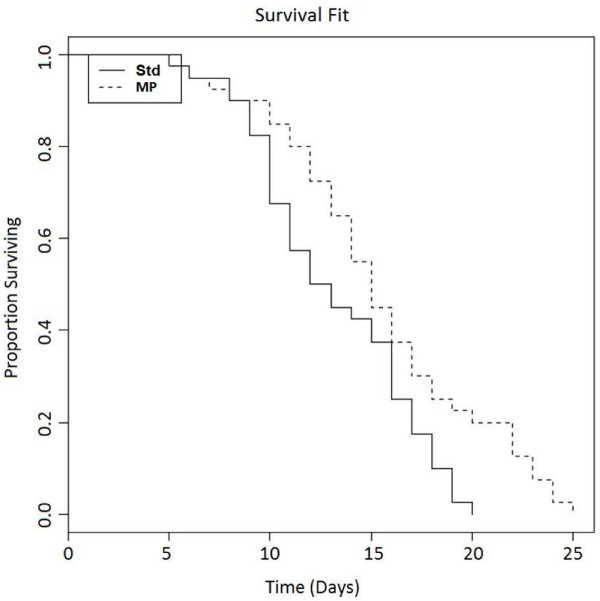
**MP fed *C. elegans *worms have a weak but significant increase in life-span**. -- represents survival of wild-type (N2) animals fed with standard agar; --- represents survival of wild-type animals fed with agar containing 20 μg/ml peptides. Control: n = 40, m = 13; Milk peptides (MP): n = 40, m = 15 (P = 0.0136). n represents the number of animals observed in each experiment. m represents the median adult life span.

A proprietary MP mixture was screened for tyrosine kinase inhibitory activity. Notably, this particular peptide mixture's IC_50 _for EGFR, VEGFR2, and IR are 9.85 μM, 7.7 μM, and 6.18 μM respectively (Figure 1). *In vitro*, the peptide mixture at 0.68 μg/μl causes cell death in HT-29 human colon cancer cell line; whereas commercial hydrolyzed whey powder did not cause cell death (Figure 2). Hoechst dye staining showed the cell death is accompanied with nuclear condensation and DNA fragmentation, suggesting an apoptotic cell death, and the apoptotic death is dose dependent (data not shown). *In vivo, C.elegans *worms were used to test the impact of MP on lifespan. Wild type L4 larval N2 worms were fed with or without the MP. In the group that was fed with agar containing 20 μg/ml peptides, the median survival where 50% worms were alive was Day 15, compared to Day 13. The last worm in the controlled group died at Day 20, in contrast to Day 25 in the experimental group fed with agar containing milk peptides (Figure 3). Statistical analysis of N2 standard vs.MP fed worms showed a value of 6.1 (p = 0.014) from χ^2 ^test, indicating a weak but significant statistical significance. Lower concentrations of peptides did not yield significant lifespan expansion (data not shown).

### Clinical Safety Study Findings

No significant MANOVA or univariate ANOVA group × time effects were seen in energy intake, macronutrient intake, or body composition between groups. However, univariate ANOVA analysis revealed small albeit significant differences in changes in fat mass (P -0.23 ± 1.3; MP 0.44 ± 1.0 kg, p = 0.04) and body fat percentage (P -0.57 ± 1.6; MP 0.2 ± 1.0%, p = 0.03) over time between groups. Resting heart rate decreased to a greater degree in the P group (P -5.4 ± 7; MP -1 ± 7 beats/min, p = 0.01) with no differences observed between groups in resting systolic or diastolic blood pressure.

**Figure 4:**
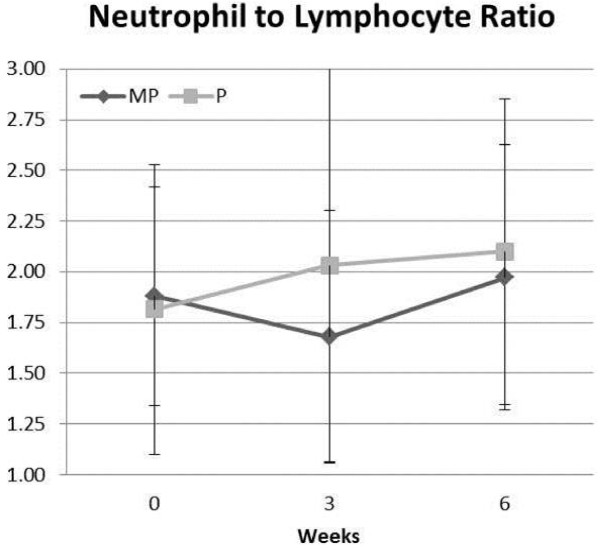
**Neutrophil to lymphocyte ratio values observed for the placebo (P) and milk protein (MP) groups over time**.

**Figure 5:**
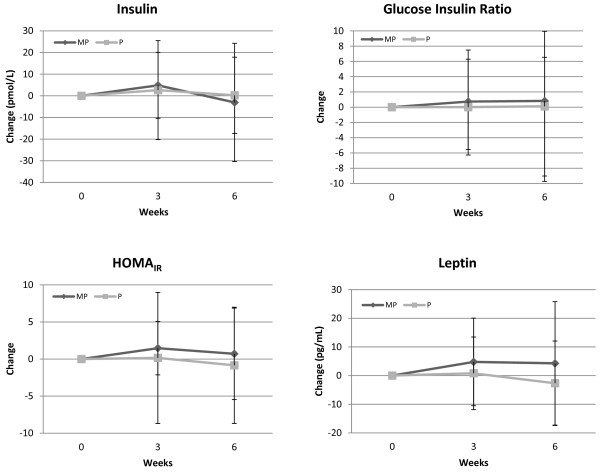
**Change in hormonal responses from baseline observed for the placebo (P) and milk protein (MP) groups**. HOMAIR represents homeostatic model assessment of insulin resistance.

Whole blood analysis revealed no overall MANOVA group × time effects (p = 0.60) among white blood cells, neutrophils, lymphocytes, monocytes, eosinphils, basophils, red blood cells, hemoglobin, hematocrit, mean cell volume, or mean corpuscle hemoglobin count. A significant univariate ANOVA quadratic interaction affect (p = 0.05) was observed among groups in the neutrophil to lymphocyte ratio (Figure 4). Serum analysis revealed no overall MANOVA or univariate group × time effects among triglycerides, total cholesterol, high density lipoproteins, low density lipoproteins, or uric acid; creatine kinase, lactate dehydrogenase, aspartate amino transaminase, alanine amino transaminase, gamma glutamyl transaminase, or alkaline phosphatase; and, blood urea nitrogen (BUN), creatinine, BUN to creatinine ratio, calcium, total protein, albumin, or total bilirubin. Figure 5 presents changes in hormonal responses observed during the study. Although no statistically significant interactions were observed, some potentially interesting trends were observed which warrant further investigation.

**Figure 6:**
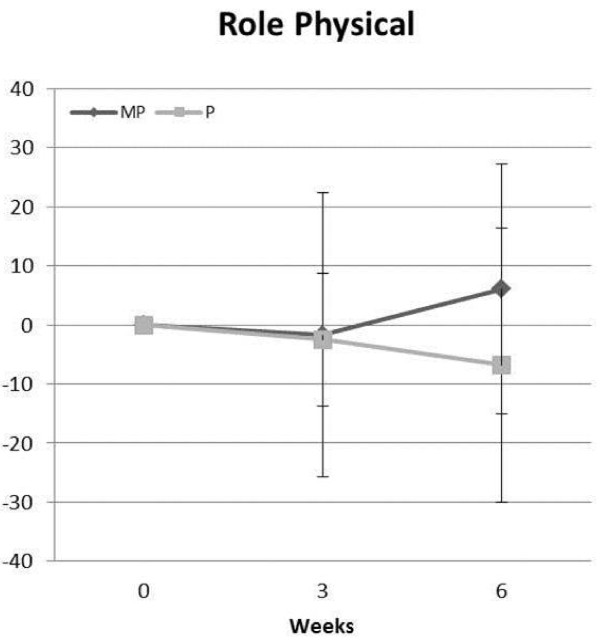
**Comparison between placebo and MP group in role physical measure of quality of life**.

MANOVA analysis indicated that a group × time trend (p = 0.06) was observed between groups in QOL indices. As Figure 6 presents, univariate ANOVA analysis revealed that participants in the MP group observed a significantly greater increase (p = 0.01) in role physical (i.e., ability to work and perform daily activities). No significant side effects were observed for frequency of dizziness, racing heart rate, skip heart beats, shortness of breath, nervousness, or blurred vision. Moreover, no subject was medically removed from the study or was referred for medical consultation.

## Discussion

Milk represents a unique source of nutrients and biologically active components that act in synergy, as well as independently. Emerging evidence indicates that the protein component of milk represents a variety of biologically active proteins/peptides that function as anti-hypertensive agents, antimicrobial factors, food intake modifiers and immune regulatory factors [8,9]. Interestingly, many bioactive peptides are inactive within their parent milk proteins, and upon release during digestion or food processing, they may act as regulatory compounds with hormone-like activity [10,11]. Additionally, there are increasing studies showing that bioactive milk peptides can be absorbed intact from the intestinal lumen into the blood circulation - these may thus serve as novel functional food ingredients or pharmaceutical agents [12-15].

Using a proprietary enzyme digestion and buffer isolation method, we purified a group of peptides from the whey fraction of regular cow's milk and screened against a panel of kinases. In particular, this milk peptide mixture shows inhibitory effects against EGFR, VEGFR2, and IR. EGFR is often overexpressed in non-small cell lung cancer (NSCLC) and a variety of common solid tumors. EGFR signaling is generally associated with cancer invasion, metastasis, chemotherapy resistance, and poor prognosis [16,17]. It has also been reported that inhibition of EGFR may lead to apoptosis in certain cancer cell lines [18]. Gefitinib (*Iressa^®^, AstraZeneca plc, London, UK*) and Erlotinib (*Tarceva^®^*, *Genentech, Inc., San Francisco, CA*) are examples of anti-cancer drugs targeting EGFR-TK. Interestingly, MP mixture is also able to cause significant cell death in HT29 colon cancer cells, whereas neither commercially available hydrolyzed whey proteins nor whole milk proteins exhibited the same property. This may be because most commercial hydrolyzed whey proteins contained too small amount of bioactive peptides or the process of spray drying deactivated the activity.

This novel MP mixture also inhibits insulin receptor signaling. Interestingly, mutations in *daf-2*, a gene that encodes an insulin-like receptor in *C. elegans *worm, have been shown to double the lifespan of the worms [19]. The gene is known to regulate reproductive development, ageing, resistance to oxidative stress, thermotolerance, resistance to hypoxia and also resistance to bacterial pathogens [20]. Therefore, we next tested this unique MP mixture effect on the lifespan of *C. elegans *worms. Statistical analysis of the result suggested that N2 worms fed with agar containing 20 μg/ml concentration of milk peptides increased the median lifespan by 15.4% (p = 0.014). Based on these data, we hypothesize that this milk peptide mixture may be a novel supplement ingredient for anti-aging and cancer preventive regimen.

Since MP has multi-kinase inhibitory activity in the micromolar range, we first conducted toxicology studies to measure the safety of these peptides. An acute and a 28-day sub-acute toxicology study showed no apparent adverse effects in rodents following oral administration of the peptides. Encouraged by the results, we conducted an 80 subject, double blind, placebo controlled study in healthy volunteers. The main purpose was to assess the safety of the milk peptides at three escalating dosages.

The clinical analyses showed that the MP mixture did not cause significant side effects in healthy human subjects. All blood and hormonal markers remained in normal ranges. There was a mean trend toward improving insulin sensitivity as assessed using HOMA_IR _that warrants additional study. This could potentially have beneficial effects for individuals with insulin resistance. However, these changes were not significant due to the large variability in responses observed. Additional study evaluating the effects of these milk peptides on glucose tolerance in individuals with and without insulin resistance would help elucidate this potential effect.

Another encouraging trend was the lowering of neutrophil to lymphocyte ratio (NLR). The neutrophil-lymphocyte ratio is a general marker of immunity and inflammatory status. Research has shown that elevated NLR to be a prognostic indicator of poor chemotherapy outcomes in advanced colorectal cancer and poor survival after colorectal liver metastases [21-23]. The potential impact of MP supplementation on lowering NLR could be used as a clinical nutritional intervention for these categories of cancer patients. In addition, these peptides have been shown to have *in vitro *anti-EGFR and VEGFR2 activity. EGFR is commonly elevated in many advanced cancers, and VEGFR2 is considered one of the key regulators of tumor-induced angiogenesis [24,25]. Together, this novel milk peptide mixture's anti-cancer and inflammatory reducing properties may be a safe, effective supplement to help cancer patients undergoing chemotherapy to obtain better clinical outcome.

In summary, preliminary experiments suggest that there may be some potentially beneficial applications of this novel MP mixture and that six weeks of human consumption appears to be safe. The limitation of this first clinical study is that the human subjects were healthy individuals. Additional clinical studies are currently underway to further elucidate the milk peptides' impact on the progression of cancer and quality of life in cancer patients.

## Competing interests

Ambryx Biotechnology, Inc. (*Riverside, CA*) provided funding for the clinical trial through an unrestricted grant to Baylor University when the Principal Investigator and the Exercise & Sport Nutrition Lab were affiliated with that institution. RBK, MI, MK, GH, and CR independently collected, analyzed, and interpreted the results from the clinical trial and have no financial interests concerning the outcome of this investigation. HC, OM and MT are research scientists affiliated with Ambryx Biotechnology.

## Authors' contributions

RBK served as Principal Investigator and contributed to the design of the study, statistical analysis, manuscript preparation, and procurement of external funding. MI served as the study coordinator, oversaw all testing, and assisted in data analysis. MC and GH assisted in data collection and performed whole blood, serum, and hormonal assays. CR served as the lab coordinator and supervised all data collection and assisted with data analysis. HC, OM and MT supervised the conduct of basic research experiments and consulted on the design of the clinical trial. All authors read and approved the manuscript.
